# A new structural model for NiFe hydrogenases: an unsaturated analogue of a classic hydrogenase model leads to more enzyme-like Ni—Fe distance and inter­planar fold

**DOI:** 10.1107/S2056989018010939

**Published:** 2018-08-14

**Authors:** Daniel J. Harrison, Alan J. Lough, Ulrich Fekl

**Affiliations:** a Department of Chemical and Physical Sciences, University of Toronto Mississauga, 3359 Mississauga Rd, Mississauga, Ontario, L5L 1C6, Canada; b Department of Chemistry, University of Toronto, 80 St. George Street, Toronto, Ontario, M5S 3H6, Canada

**Keywords:** crystal structure, NiFe hydrogenase, enzyme model, bioinorganic, sulfur ligand

## Abstract

The complex cation in the title compound, (carbonyl-1κ*C*)(1η^5^-penta­methyl­cyclo­penta­dien­yl)(μ-2,3,9,10-tetra­methyl-1,4,8,11-tetra­thia­undeca-2,9-diene-1,11-diido-1κ^2^ *S*,*S*′′′:2κ^4^ *S*,*S*′,*S*′′,*S*′′′)ironnickel(*Fe*—*Ni*) hexa­fluoro­phosphate, [FeNi(C_10_H_15_)(C_11_H_18_S_4_)(CO)]PF_6_ or [Ni(*L*′)FeCp*(CO)]PF_6_, is composed of the nickel complex fragment [Ni(*L*′)] coordinated as a metalloligand (using S^1^ and S^4^) to the [FeCp*(CO)]^+^ fragment, where (*L*′)^2−^ is [S—C(Me)=C(Me)—S—(CH_2_)_3_—S—C(Me)=C(Me)—S]^2−^ and where Cp*^−^ is *cyclo*-C_5_(Me)_5_^−^ (penta­methyl­cyclo­penta­dien­yl). The ratio of hexa­fluoro­phosphate anion per complex cation is 1:1. The structure at 150 K has ortho­rhom­bic (*Pbcn*) symmetry. The atoms of the complex cation are located on general positions (multiplicity = 8), whereas there are two independent hexa­fluoro­phosphate anions, each located on a twofold axis (Wyckoff position 4*c*; multiplicity = 4). The structure of the new dimetallic cation [Ni(*L*′)FeCp*(CO)]^+^ can be described as containing a three-legged piano-stool environment for iron [Cp*Fe(CO)‘S_2_’] and an approximately square-planar ‘S_4_’ environment for Ni. The NiS_2_Fe diamond-shaped substructure is notably folded at the S—S hinge: the angle between the NiS_2_ plane and the FeS_2_ plane normals is 64.85 (6)°. Largely because of this fold, the nickel–iron distance is relatively short, at 2.9195 (8) Å. The structural data for the complex cation, which contains a new unsaturated ‘S_4_’ ligand (two C=C double bonds), provide an inter­esting comparison with the known NiFe hydrogenase models containing a saturated ‘S_4_’-ligand analogue having the same number of carbon atoms in the ligand backbone, namely with the structures of [Ni(*L*)FeCp(CO)]^+^ (as the PF_6_^−^ salt, CH_2_Cl_2_ solvate) and [Ni(*L*)FeCp*(CO)]^+^ (as the PF_6_^−^ salt), where (*L*)^2−^ is [S—CH_2_—CH_2_—S—(CH_2_)_3_—S—CH_2_—CH_2_—S]^2−^ and Cp^−^ is cyclo­penta­dienyl. The saturated analogues [Ni(*L*)FeCp(CO)]^+^ and [Ni(*L*)FeCp*(CO)]^+^ have similar Ni—Fe distances: 3.1727 (6), 3.1529 (7) Å (two independent mol­ecules in the unit cell) and 3.111 (5) Å, respectively, for the two complexes, whereas [Ni(*L*′)FeCp*(CO)]^+^ described here stands out with a much shorter Ni—Fe distance [2.9196 (8) Å]. Also, [Ni(*L*)FeCp(CO)]^+^ and [Ni(*L*)FeCp*(CO)]^+^ show inter­planar fold angles that are similar between the two: 39.56 (5), 41.99 (5) (independent mol­ecules in the unit cell) and 47.22 (9) °, respectively, whereas [Ni(*L*′)FeCp*(CO)]^+^ possesses a much more pronounced fold [64.85 (6)°]. Given that larger fold angles and shorter Ni—Fe distances are considered to be structurally closer to the enzyme, unsaturation in an ‘S_4_’-ligand of the type (S—C_2_—S—C_3_—S—C_2_—S)^2−^ seems to increase structural resemblance to the enzyme for structural models of the type [Ni(‘S_4_’)FeCp^*R*^(CO)]^+^ (Cp^*R*^ = Cp or Cp*).

## Abstract 2

The new structural NiFe hydrogenase model [Ni(*L*′)FeCp*(CO)][PF_6_] [*L*′ = S—C(Me)=C(Me)—S—(CH_2_)_3_—S—C(Me)=C(Me)—S] is reported.

## Chemical context   

**Figure 1:**
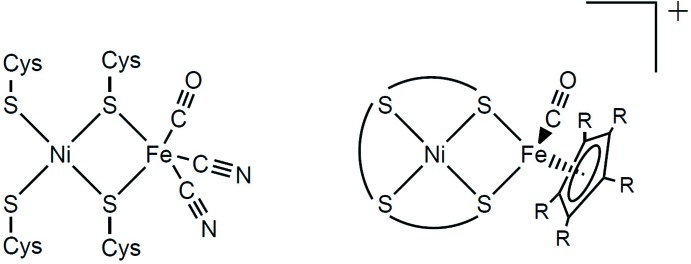
Structure of the NiFe hydrogenase active site (left) and general model of the type [Ni(‘S_4_’)Fe(Cp^*R*^)(CO)]^+^ (right; ‘S_4_’ = synthetic tetra­sulfur donor ligand).

**Figure 2:**
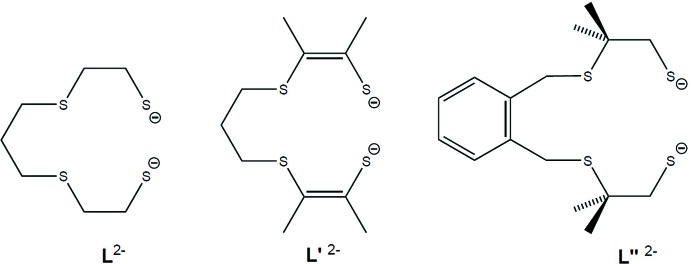
‘S_4_’ ligands used for the structurally characterized NiFe hydrogenase models of the type [Ni(‘S_4_’)Fe(Cp^*R*^)(CO)]^+^.

Since the discovery and structural elucidation of nickel–iron hydrogenases, synthetic chemists have worked towards closer and closer structural models for the NiFe hydrogen-splitting active site (Lubitz *et al.*, 2014 ▸). This active site contains two terminal sulfur donors and two bridging sulfur donors coordinated to nickel, as well as a pseudo-octa­hedal coordination sphere around iron, which is completed by cyano and carbonyl ligands (Fig. 1 ▸, left). Several closely related models of the active site have been prepared by combining an Ni(‘S_4_’) fragment (‘S_4_’ = dianionic tetra­dentate sulfur ligand) with an [FeCp^*R*^(CO)]^+^ fragment (Cp^*R*^ = Cp, C_5_H_5_ or Cp*, C_5_Me_5_), as illustrated in Fig. 1 ▸ (right) (Canaguier *et al.*, 2010 ▸; Yang *et al.*, 2015 ▸; Zhu *et al.*, 2005 ▸). These complexes have an overall mono-cationic charge, consistent with formal Ni^II^ and Fe^II^ oxidations states. The first ‘S_4_’ ligand used in this capacity featured a saturated two–three–two carbon linker, in *L* ^2−^ = [S—CH_2_—CH_2_—S—(CH_2_)_3_—S—CH_2_—CH_2_—S]^2−^ (Fig. 2 ▸, left) (Yang *et al.*, 2015 ▸; Zhu *et al.*, 2005 ▸).
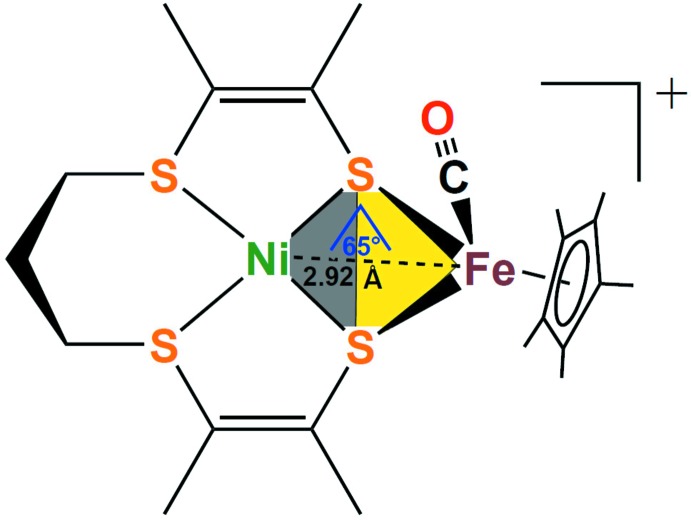


Here, we present a new [Ni(‘S_4_’)FeCp^*R*^(CO)]^+^ model based on an analogous but unsaturated ‘S_4_’ ligand, namely *L*′^2−^ = [S—C(Me)=C(Me)—S—(CH_2_)_3_—S—C(Me)=C(Me)—S]^2−^ (Fig. 2 ▸, middle), and assess the structural consequences of incorporating the unsaturated ligand. For comparison, we will also discuss a literature [Ni(‘S_4_’)Fe(Cp^*R*^)(CO)]^+^ complex in which the ‘S_4_’ ligand has a four-carbon linker in the remote portion of the backbone (*L*′′^2−^, Fig. 2 ▸, right) (Canaguier *et al.*, 2010 ▸).

## Structural commentary   

**Figure 3:**
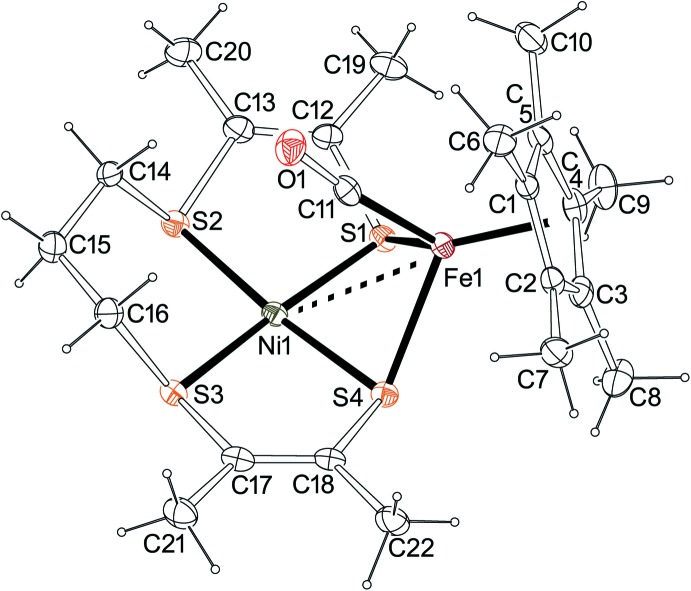
Displacement ellipsoid (30% probability) drawing for [Ni(*L*′)FeCp*(CO)]^+^, as observed in the structure of [Ni(*L*′)FeCp*(CO)][PF_6_]. Generated using *ORTEP-3 for Windows* (Farrugia, 2012 ▸).

[Ni(*L*′)FeCp*(CO)]^+^ was obtained as solvent-free crystals containing the PF_6_^−^ counter-ion. A drawing showing both cation and anion in this salt is shown below (see *Supramol­ecular features*), and the intra­molecular structural features of the cation are discussed first. The structure of [Ni(*L*′)FeCp*(CO)]^+^ is shown in Fig. 3 ▸. It contains a three-legged piano stool environment for iron and an approximately square-planar ‘S_4_’ environment for Ni (sum of bond angles around Ni1 = 359.83°). Selected metal–ligand distances are Ni1—S1 = 2.1616 (11), Ni1—S2 = 2.1530 (12), Ni1—S3 = 2.1507 (11), Ni1—S4 = 2.1563 (12) Å, and Fe1—S1 = 2.3309 (12), Fe1—S4 = 2.3602 (12), Fe1—C11 = 1.768 (5), Fe1—C1 = 2.080 (4), Fe1—C2 = 2.107 (4), Fe1—C3 = 2.126 (4), Fe1—C4 = 2.138 (4), Fe1—C5 = 2.098 (4) Å. The inter­metallic (Ni1—Fe1) distance is relatively short, *i.e.* 2.9195 (8) Å. The NiS_2_Fe diamond is markedly folded at the S—S hinge: the angle between the NiS_2_ plane and the FeS_2_ plane normals (dihedral angle; 180° − hinge angle) is 64.85 (6)°, and this fold largely accounts for the short nickel–iron distance.

In the following discussion, we compare the structural features obtained with the unsaturated ligand *L*′^2−^ with those of literature complexes using the saturated ligand *L* ^2−^. The structures of [Ni(*L*)FeCp(CO)]^+^, as the PF_6_^−^ salt/ CH_2_Cl_2_ solvate (Zhu *et al.*, 2005 ▸), and [Ni(*L*)FeCp*(CO)]^+^, as the PF_6_^−^ salt (Yang *et al.*, 2015 ▸), are known. Both saturated analogues [Ni(*L*)FeCp(CO)]^+^ and [Ni(*L*)FeCp*(CO)]^+^ show Ni—Fe distances that are similar for the two, 3.1727 (6)/3.1529 (7) Å (two independent mol­ecules in the unit cell) and 3.111 (5) Å, respectively, for the two complexes. The [Ni(*L*′)FeCp*(CO)]^+^ complex, on the other hand, has a much shorter Ni—Fe distance [2.9195 (8), see above]. Also, [Ni(*L*)FeCp(CO)]^+^ and [Ni(*L*)FeCp*(CO)]^+^ show inter­planar fold angles that are similar for the two, 39.56 (5)/41.99 (5)° (two independent mol­ecules in the unit cell) and 47.22 (9)°, respectively, while [Ni(*L*′)FeCp*(CO)]^+^ has a much larger fold angle of 64.85 (6)° (see above). The large fold angle and short Ni—Fe distance observed in the complex with the unsaturated ligand *L*′ match the structure of the enzymatic active site more closely than the angles/distances of the complexes containing the saturated ligand *L*. For eight structurally characterized enzymes, the dihedral angles range from 59 to 99° and the Ni—Fe distances range from 2.53 to 2.97 Å (one outlier being desulfovibrio fructosovorans with 46° and 3.23 Å; Zhu *et al.*, 2005 ▸). We have thus provided evidence that unsaturation in an ‘S_4_’-ligand of the type (S—C_2_—S—C_3_—S—C_2_—S)^2−^ can increase structural resemblance to the enzyme in models of the type [Ni(‘S_4_’)FeCp^*R*^(CO)]^+^. Structural similarity to the enzyme in models was, in alternative approaches, also favoured when additional donor atoms were incorporated into the ligand chain (such as ‘S_3_N_2_’) or where two bidentate chelate ligands were used instead of one large ‘S_4_’ ligand. (Zhu *et al.*, 2005 ▸) Within the context of linear ‘S_4_’ ligands, an [Ni(*L*′′)FeCp*(CO)]^+^ model with four carbon atoms, instead of three, in the remote portion of the backbone (see *L*′′^2−^ in Fig. 2 ▸, right) provided an Ni—Fe distance and fold angle very similar to those of the *L*′ analogue, of 2.9611 (8) Å and 62.48 (4)°, respectively (Canaguier *et al.*, 2010 ▸). In terms of activity, [Ni(*L*′′)FeCp*(CO)]^+^ was shown to be active as a hydrogen-production catalyst (Canaguier *et al.*, 2010 ▸), which suggests that the [Ni(*L*′)Cp^*^(CO)]^+^ complex, with the unsat­urated ‘S_4_’ ligand *L*′, might warrant deeper investigation. We conclude that the introduction of unsaturation in the ‘S_4_’ ligand led to a better structural model relative to the unsaturated ligand, highlighting a new variant of the classic [Ni(’S_4_’)FeCp^*R*^(CO)]^+^-type hydrogenase model.

## Supra­molecular features   

**Figure 4:**
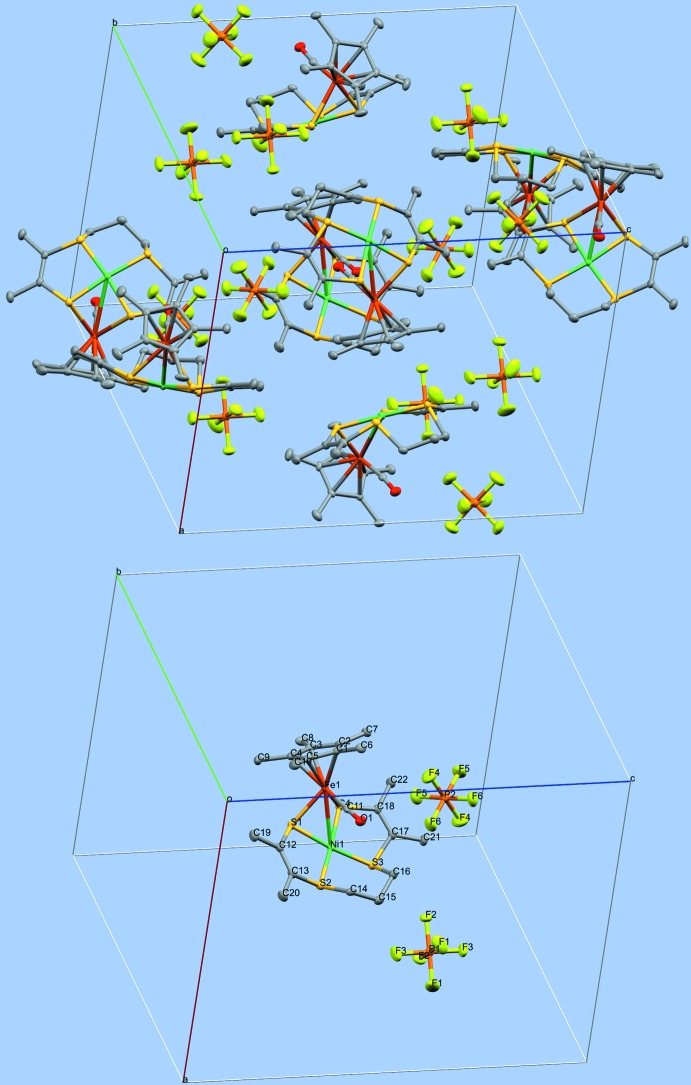
Drawings for packing (top) and labeling (bottom) of all non-H atoms in [Ni(*L*′)FeCp*(CO)][PF_6_]. Generated using *Mercury* (Macrae *et al.*, 2006 ▸). For the anion in the bottom part, generic atom labels without symmetry codes have been used.

The structure results from packing of discrete cations [Ni(*L*′)FeCp*(CO)]^+^ with hexa­fluoro­phosphate anions, without solvent mol­ecules and without any solvent-accessible void. The ratio of hexa­fluoro­phosphate anion per complex cation is 1:1. The atoms of the complex cation are situated on general positions (multiplicity = 8), whereas there are two independent hexa­fluoro­phosphate anions, each situated on a twofold axis (Wyckoff position 4*c* in *Pbcn*; multiplicity = 4). A picture of the packing is shown in Fig. 4 ▸ (top, 30% probability ellipsoids), along with labeling of all non-H atoms in the unit cell (bottom). There are no classical hydrogen bonds but there are C—H⋯F hydrogen bonds to hexa­fluoro­phosphate (C6—H6*B*⋯F4 = 2.55 Å; C15—H15*B*⋯F3^i^ = 2.55 Å; C21—H21*C*⋯F4^ii^ = 2.48 Å; C22—H22*C*⋯F1^iii^ = 2.52 Å) and a C—H⋯O short contact (C14—H14*A*⋯O1 = 2.41 Å) [symmetry codes: (i) −*x* + 2, *y*, −*z* + 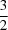; (ii) −*x* + 1, *y*, −*z* + 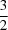; (iii) −*x* + 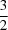, *y* + 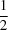, *z*].

## Database survey   

The Cambridge Crystallographic Database (version 5.39 including updates up to February 2018; Groom *et al.*, 2016 ▸) was surveyed. A search was performed aimed at finding Ni_1_Fe_1_ complexes that contain at least one (possibly substituted) cyclo­penta­dienyl unit, at least one carbonyl (CO) coordinated to iron, and a nickel center bonded to at least four sulfurs. The substructure that was used for the search contained a cyclo-C_5_ unit (any type of bond allowed), a nickel atom bonded to four sulfur atoms (any type of bond allowed), as well as an Fe–C–O unit (any type of bond for Fe—C and for C—O). Out of the six hits, RULQEV, RULQOF and RULQUL are trimetallic (instead of dimetallic) complexes (and also do not contain a cyclo­penta­dienyl but rather a saturated five-membered ring within a polycyclic structure). Since they are not very close analogues of [Ni(*L*′)FeCp*(CO)]^+^, they are not discussed further. LAZVUE (Zhu *et al.*, 2005 ▸) contains [Ni(*L*)FeCp(CO)]^+^ (as the PF_6_^−^ salt, CH_2_Cl_2_ solvate), MUDXOA (Yang *et al.*, 2015 ▸) contains [Ni(*L*)FeCp*(CO)]^+^ (as the PF_6_^−^ salt), and SUWWAJ (Canaguier *et al.*, 2010 ▸) contains [Ni(*L*′′)FeCp*(CO)]^+^ (as the BF_4_^−^ salt, CH_2_Cl_2_ solvate). These three complex cations are discussed in detail above.

## Synthesis and crystallization   

The syntheses were performed in dried solvents under an inert atmosphere (nitro­gen or argon; vacuum) using standard glove-box (MBraun) and Schlenk techniques. Deuterated NMR solvents were from Cambridge Isotopes. [Cp*Fe(CO)_2_]_2_ was acquired from Alfa Aesar. All other chemicals were obtained from Sigma–Aldrich. Photolysis was performed using a 160 W mercury vapour lamp (model: Westron Mega-Ray Self-Ballasted Zoologist).

Ni(S_2_C_2_Me_2_)_2_: This precursor for the nickel part of the complex was prepared as described in the literature (Schrauzer & Mayweg, 1965 ▸).

Ni(*L*′): Ni(*L*′), *i.e*. Ni(S—C(Me)=C(Me)—S—(CH_2_)_3_—S—C(Me)=C(Me)—S) was prepared by alkyl­ation of Na_2_[Ni(S_2_C_2_Me_2_)_2_] using 1,3-di­bromo­propane. Na_2_[Ni(S_2_C_2_Me_2_)] was prepared from Ni(S_2_C_2_Me_2_)_2_ by reduction with excess sodium in THF (344 K, 18h, in sealed vessel), until the colour had changed from deep purple to brown–yellow. The subsequent alkyl­ation of [Ni(S_2_C_2_Me_2_)]^2−^ using 1,3-di­bromo­propane was performed analogously to the procedure described by Schrauzer and co-workers for the closely related Ni(S—C(Ph)=C(Ph)—S—(CH_2_)_3_—S—C(Ph)=C(Ph)—S). (Zhang *et al.*, 1992 ▸)

[Cp*Fe(CO)_2_(NCMe)][PF_6_]: This precursor for the iron part of the complex was prepared according to the general procedure for [Cp*Fe(CO)_2_(solvent)]^+^ given by Catheline & Astruc (1984 ▸), using MeCN (acetontrile) as the solvent.

[Ni(*L*′)FeCp*(CO/NCMe)][PF_6_]: Crude [Cp*Fe(CO)_2_(NCMe)][PF_6_] (210 mg, 0.48 mmol) was combined with 6 ml of aceto­nitrile and filtered through a glass filter frit. While purging with argon, the reaction was irradiated with UV–visible light (160 W, see above) for 16 h. Under an inert atmosphere, a solution of 155 mg (0.46 mmol) of Ni(*L*′) in *ca* 7 ml of di­chloro­methane was added. The reaction mixture was heated under active argon flow to 325 K for 2 h. After cooling to room temperature, the volatiles were slowly removed under vacuum. The solid was dried under vacuum and stored in the glove-box. Yield of crude product: 253 mg (75%). ^1^H NMR (200 MHz, 298 K, CD_3_CN) δ 1.60 [*s*, (**CH_3_**)_5_C_5_]; δ 1.91 (*s*, **CH_3_**—C—S); δ 1.96 (s, **CH_3_**—C—S); δ 2.31 (*s*, *br*, **CH_3_**CN—Fe); δ 2.0–3.7 [*m*, *br*, S—(**CH_2_**)_3_—S]. Note that the sample thus prepared showed a ^1^H NMR signal for metal-coordinated aceto­nitrile. The purpose of the prolonged photolysis was to remove all CO from iron, in order to selectively prepare [Ni(*L*′)FeCp*(NCMe)][PF_6_]. However, the sample obtained appeared to be a mixture of [Ni(*L*′)FeCp*(CO)][PF_6_] and [Ni(*L*′)FeCp*(NCMe)][PF_6_] and is thus referred to as [Ni(*L*′)FeCp*(CO/NCMe)][PF_6_]. Yet, crystallization from acetone yielded exclusively [Ni(*L*′)FeCp*(CO)][PF_6_], in crystalline form.

Crystallization of [Ni(*L*′)FeCp*(CO)][PF_6_]: 11 mg of [Ni(*L*′)FeCp*(CO/NCMe)][PF_6_] were dissolved in 1.5 ml of acetone and filtered through 1 cm of Celite. Through solvent vapor diffusion, by placing the loosely capped vial into a larger vessel containing diethyl ether vapour (and some liquid), crystals of [Ni(*L*′)FeCp*(CO)][PF_6_] were grown within two days at 308 K.

## Refinement   

Crystal data, data collection and structure refinement details are summarized in Table 1 ▸. All H atoms were placed in calculated positions and included in the refinment in a riding-model approximation with C—H distances of 0.98 and 0.99 Å and *U*_iso_(H) = 1.2*U*_eq_(C) or 1.5*U*_eq_(C_meth­yl_).

## Supplementary Material

Crystal structure: contains datablock(s) I. DOI: 10.1107/S2056989018010939/zl2735sup1.cif

Structure factors: contains datablock(s) I. DOI: 10.1107/S2056989018010939/zl2735Isup2.hkl

CCDC reference: 1859284

Additional supporting information:  crystallographic information; 3D view; checkCIF report

## Figures and Tables

**Table 1:** Experimental details

Crystal data	
Chemical formula	[FeNi(C_10_H_15_)(C_11_H_18_S_4_)(CO)]PF_6_
*M*_r_	701.25
Crystal system, space group	Orthorhombic, *P* *b* *c* *n*
Temperature (K)	150
*a*, *b*, *c* (Å)	15.4081 (3), 18.3762 (3), 19.2154 (3)
*V* (Å^3^)	5440.69 (16)
*Z*	8
Radiation type	Mo *K*α
μ (mm^−1^)	1.65
Crystal size (mm)	0.20 × 0.18 × 0.12

Data collection	
Diffractometer	Nonius KappaCCD
Absorption correction	Multi-scan (*SORTAV*; Blessing, 1995 ▸)
*T*_min_, *T*_max_	0.759, 0.850
No. of measured, independent and observed [*I* > 2σ(*I*)] reflections	38285, 6224, 3874
*R*_int_	0.079
(sin θ/λ)_max_ (Å^−1^)	0.649

Refinement	
*R*[*F* ^2^ > 2σ(*F* ^2^)], *wR*(*F* ^2^), *S*	0.052, 0.148, 1.07
No. of reflections	6224
No. of parameters	335
H-atom treatment	H-atom parameters constrained
Δρ_max_, Δρ_min_ (e Å^−3^)	1.12, −0.73

Computer programs: *COLLECT* (Nonius, 1998 ▸), *DENZO-SMN* (Otwinowski & Minor, 1997 ▸), *SHELXS97* and *SHELXTL* (Sheldrick, 2008 ▸), *SHELXL2016* (Sheldrick, 2015 ▸) and *PLATON* (Spek, 2009 ▸).

## supplementary crystallographic information

### Crystal data

**Table:**

[FeNi(C_10_H_15_)(C_11_H_18_S_4_)(CO)]PF_6_	*D*_x_ = 1.712 Mg m^−^^3^
*M**_r_* = 701.25	Mo *K*α radiation, λ = 0.71073 Å
Orthorhombic, *P**b**c**n*	Cell parameters from 38285 reflections
*a* = 15.4081 (3) Å	θ = 2.6–27.5°
*b* = 18.3762 (3) Å	µ = 1.65 mm^−^^1^
*c* = 19.2154 (3) Å	*T* = 150 K
*V* = 5440.69 (16) Å^3^	Block, green
*Z* = 8	0.20 × 0.18 × 0.12 mm
*F*(000) = 2880	

### Data collection

**Table:**

Nonius KappaCCD diffractometer	6224 independent reflections
Radiation source: fine-focus sealed tube	3874 reflections with *I* > 2σ(*I*)
Detector resolution: 9 pixels mm^-1^	*R*_int_ = 0.079
φ scans and ω scans with κ offsets	θ_max_ = 27.5°, θ_min_ = 2.6°
Absorption correction: multi-scan (SORTAV; Blessing, 1995)	*h* = −19→19
*T*_min_ = 0.759, *T*_max_ = 0.850	*k* = −23→23
38285 measured reflections	*l* = −24→24

### Refinement

**Table:**

Refinement on *F*^2^	0 restraints
Least-squares matrix: full	Hydrogen site location: inferred from neighbouring sites
*R*[*F*^2^ > 2σ(*F*^2^)] = 0.052	H-atom parameters constrained
*wR*(*F*^2^) = 0.148	*w* = 1/[σ^2^(*F*_o_^2^) + (0.0765*P*)^2^ + 2.0266*P*] where *P* = (*F*_o_^2^ + 2*F*_c_^2^)/3
*S* = 1.07	(Δ/σ)_max_ = 0.002
6224 reflections	Δρ_max_ = 1.12 e Å^−^^3^
335 parameters	Δρ_min_ = −0.73 e Å^−^^3^

### Special details

**Table:**

Geometry. All esds (except the esd in the dihedral angle between two l.s. planes) are estimated using the full covariance matrix. The cell esds are taken into account individually in the estimation of esds in distances, angles and torsion angles; correlations between esds in cell parameters are only used when they are defined by crystal symmetry. An approximate (isotropic) treatment of cell esds is used for estimating esds involving l.s. planes.

### Fractional atomic coordinates and isotropic or equivalent isotropic displacement parameters (Å^2^)

**Table:**

	*x*	*y*	*z*	*U*_iso_*/*U*_eq_	
Ni1	0.74296 (4)	0.67056 (3)	0.52192 (3)	0.02311 (16)	
Fe1	0.56172 (4)	0.71009 (3)	0.49879 (3)	0.02225 (17)	
S1	0.68117 (7)	0.70012 (6)	0.42497 (5)	0.0256 (3)	
S2	0.79921 (7)	0.57496 (6)	0.47542 (5)	0.0281 (3)	
S3	0.79344 (7)	0.64527 (6)	0.62343 (5)	0.0268 (3)	
S4	0.67593 (7)	0.76476 (6)	0.56176 (5)	0.0256 (3)	
O1	0.5610 (2)	0.55947 (17)	0.54595 (17)	0.0398 (8)	
C1	0.4278 (3)	0.7053 (2)	0.5115 (2)	0.0248 (9)	
C2	0.4585 (3)	0.7723 (2)	0.5392 (2)	0.0265 (10)	
C3	0.4983 (3)	0.8115 (2)	0.4831 (2)	0.0303 (10)	
C4	0.4907 (3)	0.7698 (2)	0.4222 (2)	0.0316 (10)	
C5	0.4483 (3)	0.7029 (2)	0.4388 (2)	0.0276 (10)	
C6	0.3751 (3)	0.6504 (2)	0.5513 (2)	0.0353 (11)	
H6A	0.313257	0.661374	0.545916	0.053*	
H6B	0.390791	0.652574	0.600690	0.053*	
H6C	0.387078	0.601560	0.533256	0.053*	
C7	0.4426 (3)	0.7979 (3)	0.6122 (2)	0.0357 (11)	
H7A	0.380057	0.803763	0.619663	0.054*	
H7B	0.471790	0.844604	0.619491	0.054*	
H7C	0.465444	0.761895	0.645062	0.054*	
C8	0.5361 (3)	0.8871 (2)	0.4870 (3)	0.0452 (13)	
H8A	0.492333	0.922612	0.472476	0.068*	
H8B	0.586638	0.890341	0.456183	0.068*	
H8C	0.554009	0.897320	0.534968	0.068*	
C9	0.5187 (4)	0.7927 (3)	0.3503 (2)	0.0456 (13)	
H9A	0.475279	0.825676	0.330445	0.068*	
H9B	0.524302	0.749593	0.320604	0.068*	
H9C	0.574773	0.817703	0.353096	0.068*	
C10	0.4183 (3)	0.6449 (3)	0.3899 (2)	0.0444 (13)	
H10A	0.355519	0.649373	0.382812	0.067*	
H10B	0.431411	0.596988	0.409703	0.067*	
H10C	0.448205	0.650298	0.345239	0.067*	
C11	0.5660 (3)	0.6194 (3)	0.5293 (2)	0.0278 (10)	
C12	0.6913 (3)	0.6181 (2)	0.3746 (2)	0.0286 (10)	
C13	0.7413 (3)	0.5637 (2)	0.3961 (2)	0.0304 (10)	
C14	0.7606 (3)	0.4963 (2)	0.5252 (2)	0.0278 (10)	
H14A	0.696377	0.496663	0.526437	0.033*	
H14B	0.779375	0.450965	0.501772	0.033*	
C15	0.7957 (3)	0.4971 (2)	0.5998 (2)	0.0358 (11)	
H15A	0.786109	0.448644	0.620841	0.043*	
H15B	0.859172	0.505579	0.598102	0.043*	
C16	0.7548 (3)	0.5546 (2)	0.6466 (2)	0.0328 (11)	
H16A	0.769533	0.544153	0.695734	0.039*	
H16B	0.690822	0.552699	0.641829	0.039*	
C17	0.7331 (3)	0.7017 (2)	0.6814 (2)	0.0292 (10)	
C18	0.6833 (3)	0.7532 (2)	0.6539 (2)	0.0267 (10)	
C19	0.6439 (3)	0.6191 (3)	0.3059 (2)	0.0397 (12)	
H19A	0.673889	0.587331	0.272713	0.060*	
H19B	0.642695	0.668917	0.287647	0.060*	
H19C	0.584335	0.601759	0.312578	0.060*	
C20	0.7620 (3)	0.4947 (3)	0.3569 (2)	0.0411 (12)	
H20A	0.733297	0.495786	0.311395	0.062*	
H20B	0.741350	0.452654	0.383458	0.062*	
H20C	0.824947	0.490988	0.350310	0.062*	
C21	0.7475 (3)	0.6871 (3)	0.7574 (2)	0.0378 (12)	
H21A	0.737458	0.731855	0.783949	0.057*	
H21B	0.807296	0.670606	0.764737	0.057*	
H21C	0.707132	0.649328	0.773176	0.057*	
C22	0.6369 (3)	0.8090 (3)	0.6972 (2)	0.0381 (11)	
H22A	0.676793	0.828725	0.732196	0.057*	
H22B	0.587285	0.786252	0.720573	0.057*	
H22C	0.616285	0.848535	0.667210	0.057*	
P1	1.000000	0.50751 (9)	0.750000	0.0338 (4)	
P2	0.500000	0.56829 (10)	0.750000	0.0361 (4)	
F1	0.9262 (3)	0.44789 (18)	0.7508 (2)	0.0895 (14)	
F2	0.92696 (19)	0.56827 (15)	0.75048 (16)	0.0518 (8)	
F3	0.9995 (2)	0.5074 (2)	0.83285 (14)	0.0700 (10)	
F4	0.4168 (2)	0.5668 (2)	0.7027 (2)	0.0808 (11)	
F5	0.5402 (3)	0.62895 (18)	0.70149 (18)	0.0727 (10)	
F6	0.5418 (2)	0.50624 (17)	0.70251 (16)	0.0670 (10)	

### Atomic displacement parameters (Å^2^)

**Table:**

	*U*^11^	*U*^22^	*U*^33^	*U*^12^	*U*^13^	*U*^23^
Ni1	0.0223 (3)	0.0268 (3)	0.0202 (3)	−0.0012 (2)	−0.0002 (2)	−0.0010 (2)
Fe1	0.0216 (4)	0.0250 (3)	0.0201 (3)	0.0001 (3)	0.0002 (2)	0.0023 (2)
S1	0.0247 (6)	0.0316 (6)	0.0204 (5)	−0.0003 (5)	0.0005 (4)	0.0000 (4)
S2	0.0229 (6)	0.0327 (6)	0.0286 (6)	0.0013 (5)	0.0008 (4)	−0.0049 (4)
S3	0.0261 (6)	0.0303 (6)	0.0242 (5)	−0.0011 (5)	−0.0038 (4)	−0.0004 (4)
S4	0.0269 (6)	0.0269 (5)	0.0231 (5)	−0.0013 (5)	−0.0007 (4)	−0.0010 (4)
O1	0.036 (2)	0.0321 (18)	0.051 (2)	−0.0015 (15)	0.0016 (16)	0.0102 (15)
C1	0.021 (2)	0.028 (2)	0.025 (2)	0.0042 (18)	−0.0034 (17)	0.0000 (17)
C2	0.022 (2)	0.030 (2)	0.027 (2)	0.0050 (18)	−0.0023 (18)	0.0014 (18)
C3	0.018 (2)	0.029 (2)	0.044 (3)	0.0021 (19)	0.001 (2)	0.004 (2)
C4	0.025 (3)	0.043 (3)	0.026 (2)	0.009 (2)	0.0030 (19)	0.011 (2)
C5	0.020 (2)	0.039 (3)	0.024 (2)	0.006 (2)	−0.0028 (17)	−0.0011 (18)
C6	0.027 (3)	0.034 (2)	0.046 (3)	−0.007 (2)	0.003 (2)	0.003 (2)
C7	0.031 (3)	0.045 (3)	0.030 (2)	0.005 (2)	0.000 (2)	−0.010 (2)
C8	0.028 (3)	0.028 (2)	0.079 (4)	−0.006 (2)	0.007 (3)	0.010 (2)
C9	0.041 (3)	0.060 (3)	0.036 (3)	0.014 (3)	0.009 (2)	0.021 (2)
C10	0.036 (3)	0.057 (3)	0.040 (3)	0.007 (3)	−0.013 (2)	−0.009 (2)
C11	0.017 (2)	0.039 (3)	0.027 (2)	0.001 (2)	0.0023 (17)	0.001 (2)
C12	0.027 (3)	0.033 (2)	0.025 (2)	−0.002 (2)	0.0040 (18)	−0.0060 (18)
C13	0.029 (3)	0.039 (3)	0.024 (2)	0.001 (2)	0.0034 (19)	−0.0076 (19)
C14	0.023 (2)	0.023 (2)	0.037 (2)	0.0006 (18)	−0.0029 (19)	−0.0038 (18)
C15	0.034 (3)	0.032 (2)	0.042 (3)	0.001 (2)	−0.006 (2)	0.004 (2)
C16	0.037 (3)	0.031 (2)	0.031 (2)	0.000 (2)	−0.003 (2)	0.0036 (19)
C17	0.029 (3)	0.035 (2)	0.023 (2)	−0.008 (2)	−0.0010 (19)	−0.0039 (19)
C18	0.028 (3)	0.030 (2)	0.021 (2)	−0.007 (2)	−0.0012 (18)	−0.0052 (17)
C19	0.041 (3)	0.052 (3)	0.026 (2)	−0.009 (3)	−0.002 (2)	−0.006 (2)
C20	0.046 (3)	0.045 (3)	0.033 (2)	−0.001 (2)	0.003 (2)	−0.014 (2)
C21	0.044 (3)	0.045 (3)	0.025 (2)	−0.003 (2)	−0.005 (2)	0.002 (2)
C22	0.039 (3)	0.048 (3)	0.027 (2)	0.003 (2)	−0.005 (2)	−0.010 (2)
P1	0.0388 (11)	0.0305 (9)	0.0321 (9)	0.000	−0.0043 (7)	0.000
P2	0.0311 (10)	0.0423 (10)	0.0350 (9)	0.000	−0.0006 (8)	0.000
F1	0.112 (4)	0.055 (2)	0.102 (3)	−0.046 (2)	0.036 (3)	−0.027 (2)
F2	0.0310 (18)	0.0563 (18)	0.068 (2)	0.0122 (14)	0.0023 (14)	0.0122 (15)
F3	0.060 (2)	0.115 (3)	0.0349 (16)	0.010 (2)	−0.0033 (15)	0.0145 (17)
F4	0.061 (2)	0.090 (3)	0.092 (3)	−0.011 (2)	−0.039 (2)	0.017 (2)
F5	0.090 (3)	0.0526 (19)	0.075 (2)	−0.0221 (19)	0.016 (2)	0.0109 (17)
F6	0.089 (3)	0.061 (2)	0.0507 (18)	0.0087 (19)	0.0156 (18)	−0.0130 (15)

### Geometric parameters (Å, º)

**Table:**

Ni1—S3	2.1507 (11)	C10—H10A	0.9800
Ni1—S2	2.1530 (12)	C10—H10B	0.9800
Ni1—S4	2.1563 (12)	C10—H10C	0.9800
Ni1—S1	2.1616 (11)	C12—C13	1.328 (6)
Ni1—Fe1	2.9195 (8)	C12—C19	1.510 (6)
Fe1—C11	1.768 (5)	C13—C20	1.509 (6)
Fe1—C1	2.080 (4)	C14—C15	1.532 (6)
Fe1—C5	2.098 (4)	C14—H14A	0.9900
Fe1—C2	2.107 (4)	C14—H14B	0.9900
Fe1—C3	2.126 (4)	C15—C16	1.524 (6)
Fe1—C4	2.138 (4)	C15—H15A	0.9900
Fe1—S1	2.3309 (12)	C15—H15B	0.9900
Fe1—S4	2.3602 (12)	C16—H16A	0.9900
S1—C12	1.798 (4)	C16—H16B	0.9900
S2—C13	1.778 (4)	C17—C18	1.329 (6)
S2—C14	1.833 (4)	C17—C21	1.501 (6)
S3—C17	1.783 (5)	C18—C22	1.503 (6)
S3—C16	1.825 (4)	C19—H19A	0.9800
S4—C18	1.786 (4)	C19—H19B	0.9800
O1—C11	1.149 (5)	C19—H19C	0.9800
C1—C2	1.423 (6)	C20—H20A	0.9800
C1—C5	1.432 (6)	C20—H20B	0.9800
C1—C6	1.504 (6)	C20—H20C	0.9800
C2—C3	1.434 (6)	C21—H21A	0.9800
C2—C7	1.499 (6)	C21—H21B	0.9800
C3—C4	1.404 (6)	C21—H21C	0.9800
C3—C8	1.508 (6)	C22—H22A	0.9800
C4—C5	1.429 (6)	C22—H22B	0.9800
C4—C9	1.507 (6)	C22—H22C	0.9800
C5—C10	1.493 (6)	P1—F1^i^	1.579 (3)
C6—H6A	0.9800	P1—F1	1.579 (3)
C6—H6B	0.9800	P1—F2	1.585 (3)
C6—H6C	0.9800	P1—F2^i^	1.585 (3)
C7—H7A	0.9800	P1—F3	1.592 (3)
C7—H7B	0.9800	P1—F3^i^	1.592 (3)
C7—H7C	0.9800	P2—F4^ii^	1.572 (3)
C8—H8A	0.9800	P2—F4	1.572 (3)
C8—H8B	0.9800	P2—F5^ii^	1.580 (3)
C8—H8C	0.9800	P2—F5	1.580 (3)
C9—H9A	0.9800	P2—F6^ii^	1.596 (3)
C9—H9B	0.9800	P2—F6	1.596 (3)
C9—H9C	0.9800		
			
S3—Ni1—S2	93.13 (4)	H8A—C8—H8B	109.5
S3—Ni1—S4	91.41 (4)	C3—C8—H8C	109.5
S2—Ni1—S4	174.38 (5)	H8A—C8—H8C	109.5
S3—Ni1—S1	174.42 (5)	H8B—C8—H8C	109.5
S2—Ni1—S1	91.42 (4)	C4—C9—H9A	109.5
S4—Ni1—S1	83.87 (4)	C4—C9—H9B	109.5
S3—Ni1—Fe1	122.53 (4)	H9A—C9—H9B	109.5
S2—Ni1—Fe1	121.66 (4)	C4—C9—H9C	109.5
S4—Ni1—Fe1	52.85 (3)	H9A—C9—H9C	109.5
S1—Ni1—Fe1	52.04 (3)	H9B—C9—H9C	109.5
C11—Fe1—C1	87.63 (18)	C5—C10—H10A	109.5
C11—Fe1—C5	98.85 (18)	C5—C10—H10B	109.5
C1—Fe1—C5	40.08 (15)	H10A—C10—H10B	109.5
C11—Fe1—C2	114.69 (18)	C5—C10—H10C	109.5
C1—Fe1—C2	39.73 (15)	H10A—C10—H10C	109.5
C5—Fe1—C2	66.92 (16)	H10B—C10—H10C	109.5
C11—Fe1—C3	152.97 (19)	O1—C11—Fe1	173.2 (4)
C1—Fe1—C3	66.26 (16)	C13—C12—C19	124.2 (4)
C5—Fe1—C3	66.07 (17)	C13—C12—S1	120.9 (3)
C2—Fe1—C3	39.61 (16)	C19—C12—S1	114.7 (3)
C11—Fe1—C4	137.02 (19)	C12—C13—C20	126.9 (4)
C1—Fe1—C4	66.05 (16)	C12—C13—S2	118.0 (3)
C5—Fe1—C4	39.42 (17)	C20—C13—S2	114.8 (3)
C2—Fe1—C4	65.71 (16)	C15—C14—S2	111.4 (3)
C3—Fe1—C4	38.43 (17)	C15—C14—H14A	109.3
C11—Fe1—S1	95.64 (14)	S2—C14—H14A	109.3
C1—Fe1—S1	148.36 (12)	C15—C14—H14B	109.3
C5—Fe1—S1	108.58 (12)	S2—C14—H14B	109.3
C2—Fe1—S1	149.63 (12)	H14A—C14—H14B	108.0
C3—Fe1—S1	110.20 (12)	C16—C15—C14	114.4 (4)
C4—Fe1—S1	91.47 (12)	C16—C15—H15A	108.7
C11—Fe1—S4	101.72 (14)	C14—C15—H15A	108.7
C1—Fe1—S4	134.25 (11)	C16—C15—H15B	108.7
C5—Fe1—S4	158.40 (12)	C14—C15—H15B	108.7
C2—Fe1—S4	98.20 (12)	H15A—C15—H15B	107.6
C3—Fe1—S4	92.43 (13)	C15—C16—S3	110.7 (3)
C4—Fe1—S4	121.08 (13)	C15—C16—H16A	109.5
S1—Fe1—S4	75.92 (4)	S3—C16—H16A	109.5
C11—Fe1—Ni1	71.28 (14)	C15—C16—H16B	109.5
C1—Fe1—Ni1	157.04 (11)	S3—C16—H16B	109.5
C5—Fe1—Ni1	149.87 (12)	H16A—C16—H16B	108.1
C2—Fe1—Ni1	143.21 (12)	C18—C17—C21	126.8 (4)
C3—Fe1—Ni1	132.80 (12)	C18—C17—S3	117.8 (3)
C4—Fe1—Ni1	136.25 (12)	C21—C17—S3	115.3 (3)
S1—Fe1—Ni1	46.99 (3)	C17—C18—C22	122.7 (4)
S4—Fe1—Ni1	46.74 (3)	C17—C18—S4	121.1 (3)
C12—S1—Ni1	102.40 (15)	C22—C18—S4	116.0 (3)
C12—S1—Fe1	117.49 (15)	C12—C19—H19A	109.5
Ni1—S1—Fe1	80.97 (4)	C12—C19—H19B	109.5
C13—S2—C14	101.1 (2)	H19A—C19—H19B	109.5
C13—S2—Ni1	104.38 (15)	C12—C19—H19C	109.5
C14—S2—Ni1	107.23 (14)	H19A—C19—H19C	109.5
C17—S3—C16	102.0 (2)	H19B—C19—H19C	109.5
C17—S3—Ni1	104.64 (15)	C13—C20—H20A	109.5
C16—S3—Ni1	107.48 (15)	C13—C20—H20B	109.5
C18—S4—Ni1	103.04 (15)	H20A—C20—H20B	109.5
C18—S4—Fe1	120.33 (15)	C13—C20—H20C	109.5
Ni1—S4—Fe1	80.41 (4)	H20A—C20—H20C	109.5
C2—C1—C5	108.6 (4)	H20B—C20—H20C	109.5
C2—C1—C6	124.7 (4)	C17—C21—H21A	109.5
C5—C1—C6	126.4 (4)	C17—C21—H21B	109.5
C2—C1—Fe1	71.2 (2)	H21A—C21—H21B	109.5
C5—C1—Fe1	70.6 (2)	C17—C21—H21C	109.5
C6—C1—Fe1	128.5 (3)	H21A—C21—H21C	109.5
C1—C2—C3	107.2 (4)	H21B—C21—H21C	109.5
C1—C2—C7	124.5 (4)	C18—C22—H22A	109.5
C3—C2—C7	128.1 (4)	C18—C22—H22B	109.5
C1—C2—Fe1	69.1 (2)	H22A—C22—H22B	109.5
C3—C2—Fe1	70.9 (2)	C18—C22—H22C	109.5
C7—C2—Fe1	129.7 (3)	H22A—C22—H22C	109.5
C4—C3—C2	108.5 (4)	H22B—C22—H22C	109.5
C4—C3—C8	125.2 (4)	F1^i^—P1—F1	92.2 (3)
C2—C3—C8	126.1 (4)	F1^i^—P1—F2	179.1 (2)
C4—C3—Fe1	71.3 (2)	F1—P1—F2	88.70 (19)
C2—C3—Fe1	69.5 (2)	F1^i^—P1—F2^i^	88.70 (19)
C8—C3—Fe1	128.5 (3)	F1—P1—F2^i^	179.11 (19)
C3—C4—C5	108.8 (4)	F2—P1—F2^i^	90.4 (2)
C3—C4—C9	126.0 (4)	F1^i^—P1—F3	90.73 (19)
C5—C4—C9	125.2 (4)	F1—P1—F3	89.21 (19)
C3—C4—Fe1	70.3 (2)	F2—P1—F3	89.51 (17)
C5—C4—Fe1	68.8 (2)	F2^i^—P1—F3	90.56 (17)
C9—C4—Fe1	128.9 (3)	F1^i^—P1—F3^i^	89.21 (19)
C4—C5—C1	107.0 (4)	F1—P1—F3^i^	90.73 (19)
C4—C5—C10	127.9 (4)	F2—P1—F3^i^	90.56 (17)
C1—C5—C10	124.6 (4)	F2^i^—P1—F3^i^	89.51 (17)
C4—C5—Fe1	71.8 (2)	F3—P1—F3^i^	179.9 (3)
C1—C5—Fe1	69.3 (2)	F4^ii^—P2—F4	178.0 (3)
C10—C5—Fe1	130.4 (3)	F4^ii^—P2—F5^ii^	89.5 (2)
C1—C6—H6A	109.5	F4—P2—F5^ii^	91.9 (2)
C1—C6—H6B	109.5	F4^ii^—P2—F5	91.9 (2)
H6A—C6—H6B	109.5	F4—P2—F5	89.5 (2)
C1—C6—H6C	109.5	F5^ii^—P2—F5	90.2 (3)
H6A—C6—H6C	109.5	F4^ii^—P2—F6^ii^	89.2 (2)
H6B—C6—H6C	109.5	F4—P2—F6^ii^	89.4 (2)
C2—C7—H7A	109.5	F5^ii^—P2—F6^ii^	90.47 (18)
C2—C7—H7B	109.5	F5—P2—F6^ii^	178.67 (19)
H7A—C7—H7B	109.5	F4^ii^—P2—F6	89.4 (2)
C2—C7—H7C	109.5	F4—P2—F6	89.2 (2)
H7A—C7—H7C	109.5	F5^ii^—P2—F6	178.66 (19)
H7B—C7—H7C	109.5	F5—P2—F6	90.47 (18)
C3—C8—H8A	109.5	F6^ii^—P2—F6	88.8 (3)
C3—C8—H8B	109.5		
			
C5—C1—C2—C3	0.0 (5)	C2—C1—C5—C10	−173.1 (4)
C6—C1—C2—C3	−174.6 (4)	C6—C1—C5—C10	1.4 (7)
Fe1—C1—C2—C3	61.0 (3)	Fe1—C1—C5—C10	125.5 (4)
C5—C1—C2—C7	174.4 (4)	C2—C1—C5—Fe1	61.4 (3)
C6—C1—C2—C7	−0.2 (7)	C6—C1—C5—Fe1	−124.2 (4)
Fe1—C1—C2—C7	−124.5 (4)	Ni1—S1—C12—C13	−10.7 (4)
C5—C1—C2—Fe1	−61.0 (3)	Fe1—S1—C12—C13	−96.8 (4)
C6—C1—C2—Fe1	124.4 (4)	Ni1—S1—C12—C19	173.1 (3)
C1—C2—C3—C4	1.0 (5)	Fe1—S1—C12—C19	86.9 (3)
C7—C2—C3—C4	−173.2 (4)	C19—C12—C13—C20	1.5 (8)
Fe1—C2—C3—C4	60.9 (3)	S1—C12—C13—C20	−174.4 (4)
C1—C2—C3—C8	176.8 (4)	C19—C12—C13—S2	174.9 (3)
C7—C2—C3—C8	2.6 (8)	S1—C12—C13—S2	−1.0 (5)
Fe1—C2—C3—C8	−123.3 (5)	C14—S2—C13—C12	123.5 (4)
C1—C2—C3—Fe1	−59.9 (3)	Ni1—S2—C13—C12	12.2 (4)
C7—C2—C3—Fe1	125.9 (5)	C14—S2—C13—C20	−62.3 (4)
C2—C3—C4—C5	−1.6 (5)	Ni1—S2—C13—C20	−173.6 (3)
C8—C3—C4—C5	−177.4 (4)	C13—S2—C14—C15	−174.9 (3)
Fe1—C3—C4—C5	58.2 (3)	Ni1—S2—C14—C15	−65.9 (3)
C2—C3—C4—C9	175.9 (4)	S2—C14—C15—C16	72.5 (4)
C8—C3—C4—C9	0.1 (7)	C14—C15—C16—S3	−73.0 (4)
Fe1—C3—C4—C9	−124.3 (5)	C17—S3—C16—C15	177.2 (3)
C2—C3—C4—Fe1	−59.7 (3)	Ni1—S3—C16—C15	67.5 (3)
C8—C3—C4—Fe1	124.4 (5)	C16—S3—C17—C18	−122.3 (4)
C3—C4—C5—C1	1.6 (5)	Ni1—S3—C17—C18	−10.4 (4)
C9—C4—C5—C1	−176.0 (4)	C16—S3—C17—C21	60.1 (4)
Fe1—C4—C5—C1	60.7 (3)	Ni1—S3—C17—C21	172.0 (3)
C3—C4—C5—C10	173.4 (4)	C21—C17—C18—C22	3.9 (7)
C9—C4—C5—C10	−4.2 (7)	S3—C17—C18—C22	−173.4 (3)
Fe1—C4—C5—C10	−127.5 (5)	C21—C17—C18—S4	178.1 (4)
C3—C4—C5—Fe1	−59.1 (3)	S3—C17—C18—S4	0.8 (5)
C9—C4—C5—Fe1	123.3 (4)	Ni1—S4—C18—C17	9.1 (4)
C2—C1—C5—C4	−1.0 (5)	Fe1—S4—C18—C17	95.5 (4)
C6—C1—C5—C4	173.5 (4)	Ni1—S4—C18—C22	−176.3 (3)
Fe1—C1—C5—C4	−62.3 (3)	Fe1—S4—C18—C22	−89.9 (3)

Symmetry codes: (i) −*x*+2, *y*, −*z*+3/2; (ii) −*x*+1, *y*, −*z*+3/2.
